# Classical Conditioning as a Distinct Mechanism of Placebo Effects

**DOI:** 10.3389/fpsyt.2019.00449

**Published:** 2019-06-25

**Authors:** Przemysław Bąbel

**Affiliations:** Pain Research Group, Institute of Psychology, Jagiellonian University, Kraków, Poland

**Keywords:** classical conditioning, nocebo effect, Pavlovian conditioning, placebo effect, response expectancy

## Abstract

Classical conditioning was suggested as a mechanism of placebo effects in the 1950s. It was then challenged by response expectancy theory, which proposed that classical conditioning is just one of the means by which expectancies are acquired and changed. According to that account, placebo effects induced by classical conditioning are mediated by expectancies. However, in most of the previous studies, either expectancies were not measured or classical conditioning was combined with verbal suggestions. Thus, on the basis of those studies, it is not possible to conclude whether expectancies are involved in placebo effects induced by pure classical conditioning. Two lines of recent studies have challenged the idea that placebo effects induced by classical conditioning are always mediated by expectancies. First, some recent studies have shown that a hidden conditioning procedure elicits both placebo analgesia and nocebo hyperalgesia, neither of which is predicted by expectancy. Second, there are studies showing that visual cues paired with pain stimuli of high or low intensity induce both placebo analgesia and nocebo hyperalgesia when they are presented subliminally without participants’ awareness. The results of both lines of studies suggest that expectancy may not always be involved in placebo effects induced by classical conditioning and that conditioning may be a distinct mechanism of placebo effects. Thus, these results support the idea that placebo effects can be learned by classical conditioning either consciously or unconsciously. However, the existing body of evidence is limited to classically conditioned placebo effects in pain, that is, placebo analgesia and nocebo hyperalgesia.

## The Origins of the Classical Conditioning Account of Placebo Effects

Classical conditioning was independently suggested as a mechanism of placebo effects for the first time in 1957 by Gliedman, Gantt, and Teitelbaum (1) and Kurland (2). It is interesting that just 2 years earlier, Beecher (3) had published his seminal paper that is now considered the starting point of scientific interest in placebo effects. Thus, classical conditioning has been regarded as a mechanism of placebo effects since the very beginning of research on placebo. However, Wickramasekera (4, 5) was the first to propose a broad and coherent theoretical account of placebo effects as conditional reflexes.

According to the classical conditioning approach, placebo is a conditioned stimulus and placebo effects are conditioned responses. The first studies in which classical conditioning with an active drug as an unconditioned stimulus was used to induce placebo effects were conducted in animals (6–8). However, in fact, Pavlov (9) was the first to describe the effects of repeated applications of active drugs that his collaborators had found. Dr. Podkopaev associated the sound of a definite pitch with the effects of a dose of apomorphine in dogs. In effect, the sound of the note alone produced all the symptoms of the drug. Similarly, when Dr. Krylov repeatedly injected morphine into dogs, he observed that the preliminaries of the injection were sufficient to produce all the symptoms of the drug. Nevertheless, these early studies started two very important lines of research, that is, studies on conditioned immunopharmacological effects (10) derived from Ader and Cohen’s (6) experiment and studies on conditioned drug tolerance (11) derived from Sigel’s (8) experiment. In both lines of research, responses to stimuli that accompany the application of pharmacologically active drugs are classically conditioned. However, these studies do not aim to explore the mechanisms of placebo effects, and they focus on conditioning of physiological responses.

Voudouris, Peck, and Coleman (12–14) developed the classical conditioning paradigm to induce placebo effects in humans. By surreptitiously pairing an inactive cream with decreasing nociceptive stimulation, they strengthened the placebo effect induced by verbal suggestion of the analgesic action of the cream (12, 13). Moreover, in spite of the fact that they had previously induced the placebo effect by verbal suggestion of the analgesic action of an inactive cream, they were subsequently able to induce the nocebo effect by pairing the same cream with increasing nociceptive stimulation (12, 13). Most importantly, they also found that placebo analgesia can be induced by classical conditioning alone (without verbal suggestions); that is, the placebo effect was found in a group that was informed that they had received an inactive cream, which was then surreptitiously paired with decreasing nociceptive stimulation (14). However, it should be noted that the cream used in these studies might have raised expectancy based on previous experiences with active treatment creams and that expectancy might have biased the results. These studies started a new line of research on placebo effects induced by classical conditioning. The aim of the paper is to briefly summarize recent findings and, based on them, draw conclusions on the differential roles of classical conditioning and expectancy in placebo effects. It should be noted that subjective responses, that is, pain, are subject to conditioning in this new line of research. Thus, this paper focuses on classical conditioning of placebo effects in pain, including placebo analgesia and nocebo hyperalgesia.

## The Classical Conditioning Account Is Challenged by Response Expectancy Theory

In the same year as the first study on classical conditioning of placebo effects in humans was published (12), Kirsch (15) published his seminal paper on response expectancy in which he proposed another account of placebo effects. His theory assumes that placebo effects result from expectancies concerning placebo intervention. Kirsch (15) highlighted that, among other processes, classical conditioning is involved in the acquisition and modification of expectancy. According to this viewpoint, classical conditioning is one of the means by which expectancies are acquired and modified; that is, the effects of conditioning are mediated by expectancy (15). In other words, there is only one mechanism of placebo effects—expectancy; classical conditioning is only a method that is used to acquire or change expectancy.

This view is reflected in the popular learning model of placebo effects proposed by Colloca and Miller (16). In this model, placebo effects result from expectancies acquired by decoding information from the psychosocial context, including conditioned stimuli, among others. Thus, according to the model, classical conditioning is a mean by which placebo effects may be induced and expectancies play a central role in the formation of placebo effects induced by classical conditioning.

It should be noted that expectancies are by definition consciously accessible (17–19). According to a recent definition, expectation is understood to mean a “conscious, conceptual belief about the future occurrence of an event” (20).

Kirsch’s (15) account of the role of expectancy in the formation of placebo effects induced by classical conditioning is based on a current view on classical conditioning, which is best summarized by Rescorla (21). This modern view differs substantially from Pavlov’s (9) account, as is well reflected in the title of Rescorla’s (21) seminal paper: “Pavlovian conditioning: It’s not what you think it is.” According to this current view, classical conditioning is not a mechanical process in which one stimulus passes control over a response from another stimulus; instead, conditioning is now seen as the learning of relations among events, which allows the organism to represent its environment. As a consequence, cognitive involvement is assumed for classical conditioning. From this perspective, conditioning produces the expectancy that certain stimuli will be followed by other stimuli, and it is this expectancy that produces the response. In other words, expectancies mediate the effects of conditioning (18).

## The Challenge Continues in Studies Contrasting Classical Conditioning and Expectancy

Kirsch (15) not only challenged the classical conditioning account of placebo effects on theoretical grounds but also conducted an empirical test of his theory. Montgomery and Kirsch (22) showed that the effects of classical conditioning on placebo analgesia induced by verbal suggestions are completely mediated by expectancies and that when participants were informed that they were undergoing the conditioning procedure (i.e., pairing placebo cream with decreasing nociceptive stimulation), the conditioning did not have an effect on placebo analgesia induced by verbal suggestions.

Montgomery and Kirsch’s (22) study together with Voudouris and collaborators’ (12–14) investigations started the conditioning versus expectancy debate, which has still not been fully resolved. The essence of this debate is whether classical conditioning is a distinct mechanism of placebo effects or the effects of conditioning are mediated by expectancy. The early stage of this debate was reviewed by Stewart-Williams and Podd (19). However, during 15 years since their seminal paper was published, new research findings have been collected that shed light on the debate.

So far, few studies have been conducted in which both classical conditioning was applied and expectancy was measured. Although most of these studies suggest that the effects of conditioning are correlated with expectancy (23–26), predicted by expectancy (27), or mediated by expectancy (22, 28–30), their results are limited to participants in whom both verbal suggestions of analgesia or hyperalgesia and classical conditioning were applied. Thus, based on these findings, one cannot draw any conclusions on the role of expectancy in placebo effects induced by pure classical conditioning. Instead, it can be concluded that expectancy is involved in the effects of conditioning on placebo effects induced by verbal suggestions.

Moreover, the sparse studies in which pure classical conditioning was applied (without verbal suggestions) and expectancy was measured usually failed to induce placebo effects (25, 31, 32), probably due to limited conditioning trials (from 12 to 30, including 6–15 in which placebo was paired with changes in nociceptive stimulation). Even if it succeeded in one study (i.e., placebo analgesia was found in the group subjected to pure conditioning), the results of regression analysis revealing the prediction of the placebo effect by expectancies were based on the results from all the study groups, including those in which verbal suggestions of analgesia were provided (33). Thus, it is not possible to conclude whether expectancies predicted placebo analgesia found in the group subjected to pure classical conditioning. Interestingly, in that study, classical conditioning produced the placebo effect, regardless of whether or not participants were informed that they were undergoing the conditioning procedure (i.e., pairing placebo cream with decreasing nociceptive stimulation) and regardless of whether they were informed that active or inactive intervention was used (in fact placebo) (33). These results contradict Montgomery and Kirsch’s (22) findings.

## Challenge Accepted: Placebo Effects Induced by Pure Classical Conditioning

Unfortunately, most of the few studies in which pure classical conditioning without verbal suggestions succeeded in inducing placebo effects did not involve the measurement of expectancy (34–36). For many years, the only study in which pure classical conditioning effectively induced the placebo effect and expectancy was measured was the one conducted by Voudouris and collaborators (14). In one of the groups, participants were informed that they were in a control group and they would receive a neutral cream. They were then subjected to conditioning procedure in which the cream was paired with decreased nociceptive stimulation without participants’ knowledge. However, in that study, expectancy was measured only once (before the pre-test), so it is impossible to determine whether the conditioning that was performed after the pre-test changed expectancies.

Recently, two lines of studies have challenged the idea that placebo effects induced by classical conditioning are always mediated by expectancies. In the first line, hidden conditioning without verbal suggestions is conducted, and expectancies are measured on a trial-by-trial basis. Conditioning procedure may be conducted in two ways: by informing or not informing participants that there is a relationship between the placebo (i.e., a conditioned stimulus) and the active drug or procedure (i.e., an unconditioned stimulus). When participants are aware of the relationship, this is referred to as open conditioning; when they are not aware of it, this is called hidden conditioning. Thus, the role of consciousness is the main difference between hidden and open conditioning.

In three recent studies, hidden conditioning was used to induce placebo analgesia (37, 38) and nocebo hyperalgesia (38, 39), and expectancies were measured on a trial-by-trial basis. These studies found that not only hidden conditioning was effective in producing placebo effects but also, primarily, expectancies predicted or mediated neither placebo analgesia nor nocebo hyperalgesia (37–39), even though conditioning had an effect on expectancies (37). Moreover, when participants were asked at the end of the study whether they had noticed the contingency between placebo stimuli and differences in pain intensity, most of them denied (37). Thus, based on these results, it seems that it is possible to induce placebo effects without the awareness of the participants.

The second line of research that sheds light on the role of expectancy in placebo effects induced by pure classical conditioning involves placebo stimuli presented subliminally without participants’ awareness. In this paradigm, clearly recognizable visual stimuli are first paired with pain stimuli of high or low intensity. After a conditioning phase is completed, the same conditioned visual cues are presented subliminally in a testing phase. It has been found that pain stimuli preceded by subliminally presented conditioned visual stimuli are rated as less or more painful depending on whether they have previously been paired with high or low pain stimuli, indicating that placebo analgesia and nocebo hyperalgesia are induced without awareness (40–44). Moreover, it has also been found that both placebo analgesia and nocebo hyperalgesia can be induced not only by conditioning of supraliminal stimuli but also by conditioning of subliminally presented stimuli (44). Placebo effects induced by conditioned stimuli presented subliminally without participants’ awareness suggest that expectancy may not have been involved in their production, which is consistent with the results from the first line of studies. Although expectancy is not measured in those studies, participants are not aware of the presented stimuli. Thus, their expectancy should not have affected the results.

It may be argued that the studies from both lines of research did not include any placebo interventions in the form of a sugar pill, fake cream, or sham electrodes. In fact, in all of those studies, visual stimuli were paired with decreasing or/and increasing pain stimulation. However, according to Miller and Kaptchuk (45), the placebo effect is not the result of a specific intervention, but it is rather produced and enhanced by the context surrounding the treatment. Thus, even if no inert treatment is administered, the so-called placebo-related effect may still be found (46).

## Conclusions

The results of both lines of studies suggest that expectancy may not be always involved in placebo effects induced by classical conditioning and that conditioning may be a distinct mechanism of placebo effects.

These findings are in line with the fact that, in some cases, classical conditioning represents an automatic process that is not mediated by cognitive expectancy (18). In fact, many phenomena could be explained by classical conditioning without cognitive mediation. They include evaluative conditioning, second-order conditioning, conditioned taste aversions and flavor preferences, conditioning with subliminally presented conditioned stimuli, conditioned immunosuppression, and conditioning in simple organisms among others (see (18) for review). Thus, only some placebo effects could be explained by classical conditioning without expectancy involvement.

However, the findings under discussion do not exclude the role of expectancy in inducing placebo effects. Expectancy ratings may not always predict placebo effects. However, pre-cognitive associations, that is, “links between events and/or objects that exist outside conscious awareness” (20), may be acquired through hidden conditioning procedures or be responsible for responses to subliminally presented conditioned stimuli. In fact, when classical conditioning is used to enhance or reduce placebo effects induced by verbal suggestions, expectancies are involved in their formation (22–30). In that case, classical conditioning is just a mean by which expectancies are acquired and modified. Moreover, expectancies might not always be easily self-reported; that is, although expectancies do exist, one might not be able to report them. However, the idea of conscious expectancies that are not self-reported should be dealt with caution as it may lead to circular reasoning (17).

These conclusions are in line with recent review (47) and previously proposed models postulating that the classical conditioning and response expectancy accounts do not exclude each other, but the range of phenomena they explain is not completely the same (19, 48). Conditioning involves either conscious learning (acquisition and modifications of expectancies) or unconscious learning (conditioning not mediated by expectancy). Expectancies can be acquired and modified by conditioning and other procedures, including verbal suggestions and observational learning. In other words, either conscious learning (expectancy and conditioning) or unconscious learning (conditioning) can be mechanisms of placebo effects. Thus, both accounts seem to be compatible rather than mutually exclusive (19, 48). From this perspective, classical conditioning is in some cases a distinct mechanism of placebo effects, and sometimes, it is just a method used to acquire or change expectancy.

Thus, the current conclusions contradict Colloca and Miller’s (16) learning model of the formation of placebo effects. They suggest that conditioned placebo and nocebo responses may not always be mediated by expectancy. It seems that Colloca and Miller’s (16) model does not explain the mechanism of all instances of placebo effects. However, future studies should answer the question under which circumstances placebo effects induced by classical conditioning are mediated by expectancy and when they are not mediated by expectancy. Previous studies in which expectancies were not involved in the induction of placebo effects by classical conditioning used visual stimuli as placebos together with a large number of conditioning trails. Thus, these two factors may be necessary to induce conditioned placebo effects that are not mediated by expectancy. So far, it seems only clear that placebo effects induced by both conditioning and verbal suggestions are mediated by expectancy. Further research is also needed to investigate the differential role of classical conditioning and expectancy in placebo effects outside pain. It would also be of interest to investigate whether all principles of classical conditioning found in studies outside the placebo research field (e.g., generalization and extinction) can be directly applied to placebo effects.

The finding that expectancy may not always be involved in placebo effects induced by classical conditioning has implications that have been discussed above, not only for placebo theory. It also has important implications for the methodology of placebo studies, that is, that expectancies should be measured in research on placebo effects when the role of expectancy is under study. Regardless of whether placebo effects were induced by classical conditioning, verbal suggestions, or both, the involvement or absence of expectancy might be postulated only when expectancy was measured. Most importantly, this fact also has implications for clinical practice. Pain can decrease or increase after negative or positive experiences that are associated with environmental stimuli. In effect, these environmental stimuli may increase or reduce pain symptoms, not only without any provided verbal suggestions, but—most importantly—without patients’ conscious awareness. Thus, pain changes can occur even when patients do not anticipate them. The decrease or increase of pain may result from uncontrollable contextual factors. Identifying the elements, that is, the conditioned stimuli that change pain experiences, could be an essential part of pain management programs. However, as significant differences between experimental and clinical settings exist, further studies are needed before translating laboratory research results into clinical practice.

## Author Contributions

The author confirms being the sole contributor of this work.

## Funding

This manuscript was prepared under grant number 2014/14/E/HS6/00415 from the National Science Centre, Poland.

## Conflict of Interest Statement

The author declares that the research was conducted in the absence of any commercial or financial relationships that could be construed as a potential conflict of interest.
